# Preparation, Characterization, and Chemically Modified Date Palm Fiber Waste Biomass for Enhanced Phenol Removal from an Aqueous Environment

**DOI:** 10.3390/ma16114057

**Published:** 2023-05-30

**Authors:** Nadavala Siva Kumar, Mohammad Asif, Anesh Manjaly Poulose, Ebrahim H. Al-Ghurabi, Shaddad S. Alhamedi, Janardhan Reddy Koduru

**Affiliations:** 1 Department of Chemical Engineering, King Saud University, P.O. Box 800, Riyadh 11421, Saudi Arabia; masif@ksu.edu.sa (M.A.); ealghurabi@ksu.edu.sa (E.H.A.-G.); shaddadalhamedi@gmail.com (S.S.A.); 2 SABIC Polymer Research Centre, Department of Chemical Engineering, King Saud University, P.O. Box 800, Riyadh 11421, Saudi Arabia; apoulose@ksu.edu.sa; 3 Department of Environmental Engineering, Kwangwoon University, Seoul 01897, Republic of Korea; redddyjchem@gmail.com

**Keywords:** adsorption, date palm fiber, phenol, kinetic and isotherm studies

## Abstract

The date palm tree is extensively cultivated in Middle Eastern countries such as Saudi Arabia, generating a large amount of waste in the form of leaves, seeds, and fibrous materials. This study examined the feasibility of using raw date palm fiber (RDPF) and NaOH chemically modified date palm fiber (NaOH–CMDPF) obtained from discarded agricultural waste for the removal of phenol in an aqueous environment. The adsorbent characterization was performed by using different techniques, i.e., particle size analysis; elemental analyzer (CHN); and BET, FTIR, and FESEM-EDX analysis. The FTIR analysis revealed the presence of various functional groups on the surface of the RDPF and NaOH–CMDPF. The results showed that chemical modification by NaOH increased the phenol adsorption capacity that was well-fitted by the Langmuir isotherm. Higher removal was obtained with NaOH–CMDPF (86%) than with the RDPF (81%). The RDPF and NaOH–CMDPF sorbents’ maximum (Q_m_) adsorption capacities were more than 45.62 mg/g and 89.67 mg/g and were comparable to the sorption capacities of various other types of agricultural waste biomass reported in the literature. The kinetic studies confirmed that the adsorption of phenol followed the pseudo-second-order kinetic process. The present study concluded that the RDPF and NaOH–CMDPF were eco-friendly and cost-effective in promoting sustainable management and the reuse of the Kingdom’s lignocellulosic fiber waste material.

## 1. Introduction

Phenol (C_6_H_5_OH) is one of the most important pollutants that is often released into wastewater via the manufacturing processes of the plastic, paint, and textile industries [1,2]. According to estimations by the United States Environmental Protection Agency (USEPA) and the Andhra Pradesh State Pollution Control Board (APSPCB), phenolic wastes released from industries such as the polymer, pharmaceutical, and petrochemical industries amounted to 56,000 tons/year in the USA and 190 tons/month in Andhra Pradesh in India [3,4]. Phenolic compounds have been enlisted by the United States Environmental Protection Agency (USEPA) and the European Union (EU) as pollutants of primary concern. This enlistment is because these chemicals are noted to be toxic and have severe short- and long-term effects on humans and animals. Phenol leads to serious health effects for humans; hence, its removal from wastewater is crucial. Effluents from such industries often contain a mixture of phenolic compounds such as phenol, nitrophenols, chlorophenols, cresol, etc. These compounds are harmful even at low concentrations. Oral ingestion of phenols, even in small amounts (1 g), is known to be lethal, with symptoms such as loss of coordination due to muscle weakness, tremors, convulsions, paralysis, and respiratory arrest [5,6].

Numerous water treatment technologies, such as photo-degradation, coagulation-flocculation, chemical oxidation, biological processes, etc., are employed for the removal of phenolic compounds [7]. Recently, the adsorption technique has emerged as a low-cost, simple, non-toxic, and effective method of treating wastewater contaminated with organic pollutants, including phenolic compounds [8]. Among the various processes, adsorption onto the surface of activated carbon (AC) appears to be the most popular and widely used method in treating high-strength and low-volume phenolic wastewater in batch and column studies. The removal of phenols by adsorption treatment is simple and easy to implement, even for large-scale operations. Nonetheless, the high cost of activated carbon is often the main factor affecting the economy of the separation process. Therefore, the focus of many researchers has been to develop unconventional adsorbents, preferably of low cost [4,9]. To this end, waste products from industries and agricultural products are promising due to their abundant availability and low cost, which means they require no regeneration after use [10]. In fact, an adsorbent can be considered low-cost if less processing is involved in its preparation and its precursor is locally available in abundance. Several studies have been conducted on the adsorption of phenols on various agricultural wastes, industrial wastes, and natural resource materials such as tamarind seed powder and tamarind nutshell [6,11], pomegranate peel ash [12], jute stick [13], wheat husk [14], olive pomace [15], de-oiled soya [16], chitin [17], olive mill waste [18], coconut shell and rubber seed coat [19,20], chitosan [21], chitosan–abrus precatorius blended beads [22], and pine cone and pine bark powder [7,23]. However, date palm, among all the agricultural wastes, can be considered one of the best choices in the context of Saudi Arabia for the removal of phenols due to its high carbon content, low price, and abundant large-scale availability [24]. Date palm stones, fibers, and leaves have been effectively employed for the removal of dyes [25,26], heavy metals [27,28,29,30,31], 2,4-dinitrophenol, and phenol [32,33,34]. Therefore, local date palm agro-waste biomass has been specifically chosen in this present work due to its abundant availability in the Kingdom of Saudi Arabia.

This work aims to assess the performance of raw and NaOH chemically modified date palm agro-waste (RDPF and CMDPF). The treatment of phenols is examined to study the influence of various operating conditions on the adsorbent, such as initial pH, contact time, sorbent dosage, and adsorbate concentration, by carrying out batch equilibrium studies. Rigorous characterization of the samples was undertaken by using a particle-size analyzer, an elemental analyzer (CHN), BET surface area, FESEM-EDX, and FTIR analysis in order to understand the correlations between the relevant properties of the adsorbent and its contaminant uptake efficacy.

## 2. Materials and Methods

### 2.1. Chemicals

Phenol (purity 99.5%) was procured from Sigma-Aldrich (St. Louis, MO, USA) and used without further purification. Its molecular formulas and weights were C_6_H_5_OH and 94.11 g/mol, respectively. To prepare stock solutions, 1.07 g of phenol was dissolved in 1000 mL of double-distilled water. The working concentration range of 50–200 mg/L required in our experiments was obtained using the stock solution. Solution pH was set with the help of 0.1 M HCl and 0.1 M NaOH solutions.

#### 2.1.1. Preparation of Date Palm Fiber Waste Biomass

Raw date palm fiber agro-waste biomass material was collected from date palm orchards around Riyadh City, Kingdom of Saudi Arabia. Raw date palm fiber was washed with tap water to remove dirt and dried in sunlight for 48 hrs. After washing, the raw fiber was chopped into lengths of 2–4 cm. The fiber was crushed by using a milling instrument and then passed through a sieve with a 0.4 mm opening. The crushed date palm fiber was then ground using a DLC multifunctional grinder and sieved. Next, the collected sieved powder was ground in a ball-milling apparatus (Fritsch, Pulverisette 7 Premium line, Idar-Oberstein, Germany) with zirconia ceramic and steel balls at 400 rpm for 24 h and POWTEQ Laboratory (Micro Ball mill, GT300, Beijing, China) micro ball-milling apparatus for 30 min at 1500 rpm. The raw ball-milled date palm fiber sample was labeled as RDPF.

#### 2.1.2. Modification of Date Palm Fiber

The RDPF biomass powder was chemically modified with sodium hydroxide (NaOH) using the method previously described by Ye et al. (2010), with minor modifications [35]. Then, 10 g of the RDPF was added to 200 mL of 1 M NaOH solution at 400 rpm, stirring for 24 h. Next, the slurry was allowed to settle for 36 h and then the mixture was heated for 2 h at 120 °C. The slurry material was filtered and washed with double distilled water until the pH of leeched water had become neutral (pH = 7) and dried in an oven at 60 °C for 24 h to reach a constant weight. Finally, the sodium hydroxide chemically modified dried sample was labeled as NaOH–CMDPF and stored in an airtight container to be used for further adsorption studies.

### 2.2. Batch Studies

The batch equilibrium experiments were carried out using amber glass reagent bottles containing a 100 mL solution of phenol with a 50–200 mg/L concentration, 0.1 g of RDPF, and NaOH–CMDPF at 30 ± 1 °C. The mixtures were continuously stirred in a water bath shaker at 175 rpm until equilibrium was reached. The pH of the solution was changed in the range of 2–10. After shaking, the suspension was filtered through Whatman grade 41 paper to obtain the supernatant solution. The residual concentrations of phenol were measured via a UV-Vis spectrophotometer (model: Shimadzu UV-1900, Tokyo, Japan) at a wavelength of 270 nm to determine the equilibrium uptake capacity of the adsorbent.

The equilibrium uptake capacity (*q_e_*, mg/g) and removal efficiency of the biosorbent were evaluated using the following equations [36]:

(1) $$q_{e}=\frac{\left(C_{0}-C_{e}\right)V}{M}$$

(2) $$Removal\;\left(\%\right)=\frac{\left(C_{0}-C_{e}\right)\times 100}{C_{0}}$$

where *C*_0_ and *C_e_* represent the initial and equilibrium phenolic concentrations (mg/L) in the aqueous medium, respectively, during the batch isotherm studies that were carried out using the *V* (L) solution containing biosorbent mass *M* (g).

The time-dependent phenol uptake during the batch kinetics studies was determined by using the following material balance:

(3) $$q_{t}=\frac{V\left(C_{0}-C_{t}\right)}{M}$$

where the transient phenolic concentration ($C_{t}$, mL/L) in the aqueous medium was monitored with respect to time *t* (min).

### 2.3. Normalized Standard Deviation

The validity of the kinetic models was analyzed by computing the normalized standard deviation, which can be mathematically represented as follows [37]:

(4) $$∆q\left(\%\right)=100\times \sqrt{\overset{N}{\underset{i=1}{\sum }}\frac{1}{\left(N-1\right)}{\left[\frac{\left(q_{i,exp}-q_{i,cal}\right)}{q_{i,exp}}\right]}^{2}}$$

where *q_i,exp_* is the experimental data while *q_i,cal_* represents the corresponding model predictions. *N* represents the number of experimental data points.

### 2.4. Chi-Square Test (χ^2^)

Another test of the model validity was determined by carrying out a Chi-square analysis, which is given as follows [38]:

(5) $$\chi^{2}=\overset{N}{\underset{i=1}{\sum }}\frac{{\left(q_{e}-q_{e,model}\right)}^{2}}{q_{e,model}}$$

where $q_{e,model}$ represents the theoretical concentration of the phenol on the biosorbent at equilibrium predicted by the model, while $q_{e}$ is the actual value achieved experimentally.

### 2.5. Characterization of RDPF and NaOH–CMDPF

The particle size was determined using a laser diffraction particle size analyzer (Shimadzu, SALD-2300, Kyoto, Japan). Particle size was identified by the light intensity distribution pattern of scattered light that was irradiated from the sample particle surface. Elemental analysis of the date palm biomass samples (C, H, N) was performed using a PerkinElmer 2400 CHNS/O series II analyzer (Norwalk, Connecticut, CT, USA), operated in CHN mode. Approximately 2 mg of each sample was used for the measurement. Each measurement was run in duplicate and the reported values are the averaged results from each set of duplicates. The specific surface area, pore volume, and average pore size were studied by the BET method. This is a very important technique for the measurement of the specific surface area of materials. The morphological studies were analyzed with a field emission scanning electron microscope (FESEM, JSM-7600F-JEOL, Tokyo, Japan) and an energy-dispersive X-ray spectroscopy (EDX) facility. The adsorbent surface functional groups were determined by Fourier transform infrared spectrophotometry (Shimadzu, IR-PRESTIGE-21, Japan) with a spectral range from 400 to 4000 cm^−1^.

## 3. Results and Discussion

### 3.1. Characterization of Date Palm Fiber Biomass

#### 3.1.1. Particle Size Data Analysis

The average particle size of the RDPF biomass samples allowed for a better insight into the size reduction and particle size distribution of both size reduction strategies, i.e., 24 h ball-milling and 30 min micro-ball-milling, shown in Supplementary Figures S1 and S2 by depicting the size distribution in terms of cumulative and differential volume percentages. The effect of 24 h ball-milling was clearly pronounced in the case of RDPF, where the initial particle size of 75–106 µm was reduced to 0.0889 µm. Conventional ball-milling was, therefore, considered effective in this case in view of the almost 99.9% size reduction rendered by this technique. However, micro-ball-milling of the DPF sample yielded an even greater size reduction with only a 30 min contact duration. The average sample size, in this case, was 0.045 µm, which was half of the size obtained with 24 h conventional ball-milling. Therefore, these results conclusively prove that micro-ball-milling is, undisputedly, an effective size reduction technique that can be used to produce nano-sized samples that are otherwise not possible with the conventional ball-milling size reduction technique.

#### 3.1.2. Elemental (C, H, N) Analysis

The elemental composition of carbon, hydrogen, and nitrogen of the raw micro-ball-milled and NaOH chemically modified date palm waste biomass is reported in Table 1. Al-Khalas date palm (*Phoenix dactylifer*) tree fiber was used in this study. Once the chemical activation of the RDPF biomass sample using NaOH had been carried out, a small decrease in the carbon content was noted, with the complete elimination of the nitrogen [39,40]. Note that there was a small increase in hydrogen, which was perhaps due to the presence of hydrogen in the –OH group owing to the NaOH-induced chemical modification of the ball-milled sample.

#### 3.1.3. BET Analysis

The BET characterization of raw date palm fiber, 24 h ball-milled, micro-ball-milled, and NaOH chemically treated biomass samples of N_2_ adsorption/desorption isotherm curves are shown in Supplementary Figures S3–S6. Conventional ball-milling led to a size reduction of RDPF from 75–106 µm to 0.089 µm, while the micro-ball-milling strategy reduced the size to 0.045 µm. The RDPF biomass BET surface area was 0.8890 m^2^/g; the pore volume and pore size were 0.0062 and 559.9 Å, respectively. However, the pore volume and pore size of the DPF unground raw samples did not show such a significant difference as that observed for the case of a specific surface area. The micro-ball-milling DPF surface area was 3.5549 m^2^/g; the pore volume and pore size were 0.0171 and 186.0 Å, respectively. The NaOH chemically modified DPF surface area was 0.04025 m^2^/g; the pore volume and pore size were 0.0062 and 588.5 Å, respectively.

#### 3.1.4. FESEM-EDX Analysis

**Figure 1:**
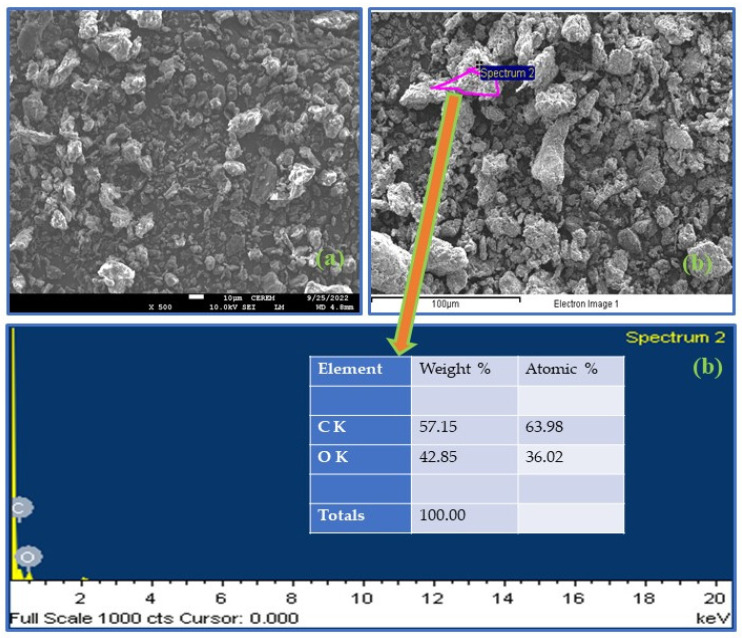
FESEM-EDX images of raw biomass (**a**) RDPF (**b**) EDX analysis.

**Figure 2:**
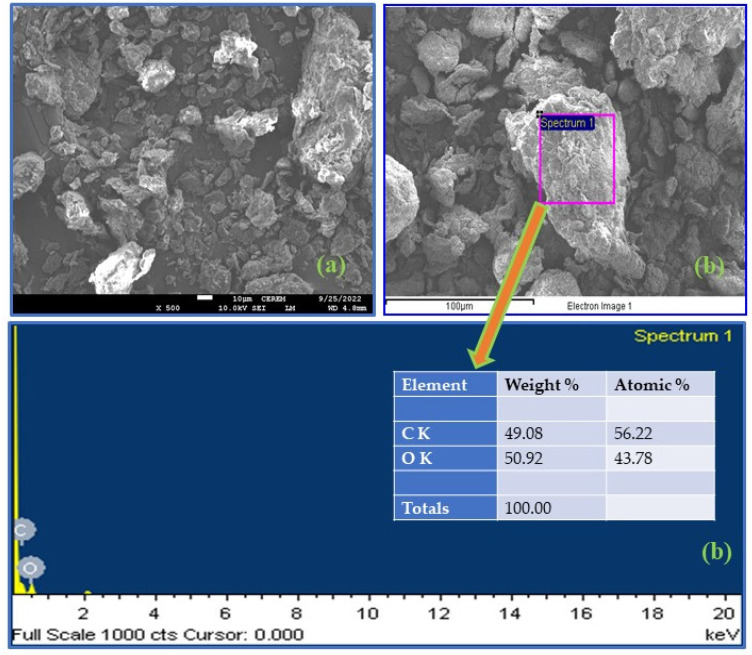
FESEM-EDX images of chemically modified biomass (**a**) NaOH-CMDPF (**b**) EDX analysis.

Field emission scanning electron microscope (FESEM) analysis was used to understand the surface morphology of the RDPF and NaOH–CMDPF, as shown in Figure 1a and Figure 2a. FESEM images clearly show that the surface of raw date palm (RDPF) was smooth compared to the surface of chemically treated biomass (NaOH–CMDPF) and had a porous nature and irregularly shaped structural particles with different size ranges of 40–500 nm. The chemically modified biomass had a rough surface that enhanced the removal of phenols from the wastewater. The ultimate composition of the RDPF and NaOH–CMDPF (C, H, N) was also confirmed by the EDX elemental (Figure 1b and Figure 2b), and the semi-quantitative analysis results were determined to be consistent with the C, H, N elemental analysis.

#### 3.1.5. FTIR Analysis

**Figure 3:**
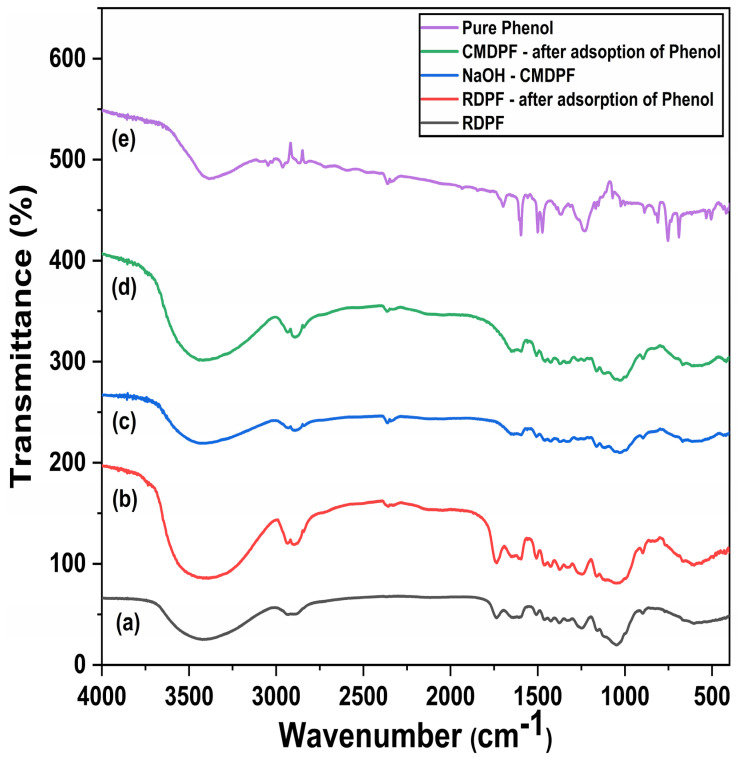
FTIR analysis of date palm fiber: (**a**) RDPF; (**b**) RDPF after adsorption of phenol; (**c**) NaOH-CMDPF; (**d**) NaOH-CMDPF after adsorption of phenol’ (**e**) pure phenol.

The FTIR spectra of the RDPF and NaOH–CMDPF biomass compositions were determined from the bands in the range of 4000–400 cm^−1^, as shown in Figure 3. The highest broadband peak at approximately 3421–3425 cm^−1^ confirmed the presence of O-H stretching and indicated the presence of alcohol groups. Figure 3 shows that the remaining peaks were C–H, C=O, N–H, –C–H, C–N, and C–Cl stretching frequencies at 2920–2933, 1733–1745, 1624–1654, 1370–1445, 1050–1250, and 603–810 cm^−1^, respectively, indicating the presence of aliphatic, carboxylic acid, amide, alkane, amine, and alkyl halide functional groups, respectively [39,41]. The vibration bands of C=O (1735 cm^−1^), C-H_2_ deformation (1346 cm^−1^), C-O-C (1180 cm^−1^), and C-O (1010 cm^−1^) stretching of primary and secondary alcohol were predictable from the cellulose, hemicellulose and C=C (1514 cm^−1^), C-C, and C-O (1245, 1065 cm^−1^) stretching frequencies of lignin [42,43]. These bands were mainly expected from waxes such as fatty acids, fatty esters, and high molecular mass aldehydes/ketones. The NaOH–CMDPF biomass spectrum peaks also confirmed the presence of O-H and C-H stretching band vibrations at 3425–3439 cm^−1^ and 2904–2925 cm^−1^, respectively. The C=C stretching, C–N, S=O, C-O, and C=C bending frequencies at 1608–1641, 1056–1068, and 663–669 cm^−1^, respectively, indicated the presence of conjugated alkene, amine, sulfoxide, and alkene functional groups, respectively.

### 3.2. Investigation of Solution pH

**Figure 4:**
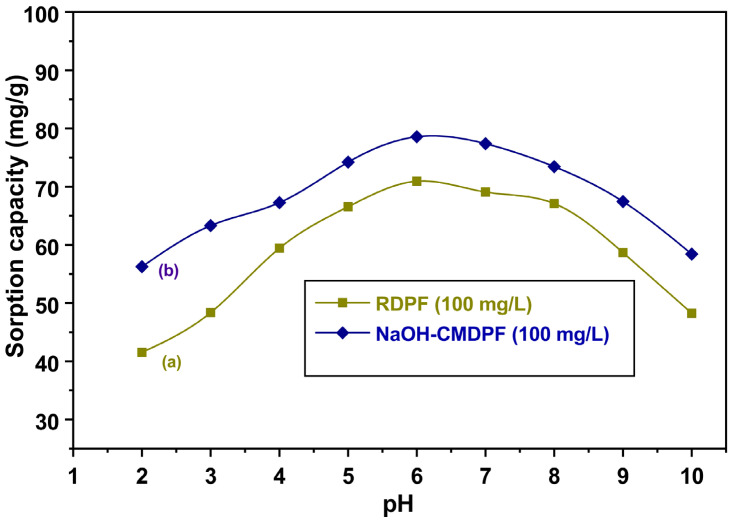
Effect of pH on phenol adsorption onto (**a**) RDPF and (**b**) NaOH-CMDPF at 30 ± 1 °C (C_0_ = 100 mg/L, contact time = 4 h, adsorbent dosage = 0.1 g, agitation rate = 175 rpm.

The sorption capacity strongly depended upon the pH of the aqueous medium. Its influence was, therefore, analyzed on the phenol uptake by varying the pH values from 2 to 10. We observed a strong correlation between the phenol uptake by the biosorbent and the solution pH. As shown in Figure 4, the phenol uptake increased from 41.54 to 70.93 mg/g and from 56.26 to 78.57 mg/g by RDPF and NaOH-CMDPF, respectively, when the solution pH was increased from 2 to 6. However, a further increase in the pH in the range of 7–10 lowered the biosorptive capacity. At pH values greater than 6, phenols mostly exist in salt forms that can easily lose their negative charge, causing difficulties with adsorption and leading to a decrease in the biosorptive capacity of the adsorbent [44]. On the other hand, the protonated phenols at lower pH values were more absorbable than their (non)-ionized counterparts. A similar trend was observed in various agricultural wastes, e.g., peanut shells, walnut shells, pumpkin seed shells, and sunflower seed hulls [38,45]. Therefore, further experiments were led at the optimal pH value of 6.

### 3.3. Influence of Sorbent Dosage

**Figure 5:**
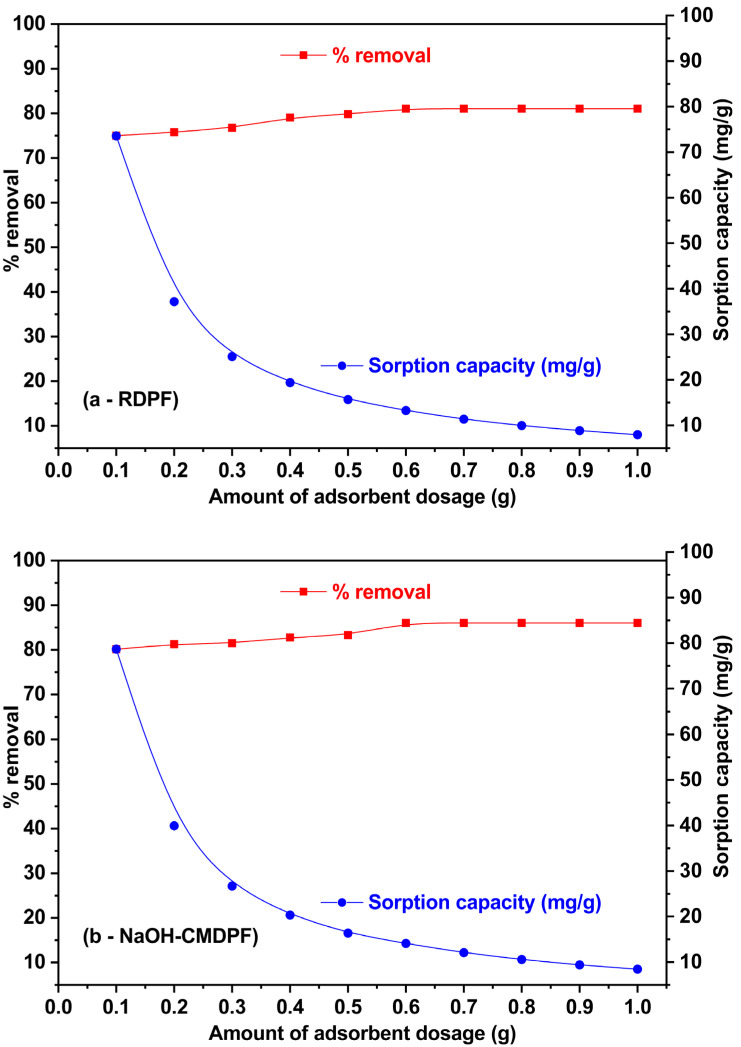
(**a**,**b**) Phenol adsorption onto (**a**) RDPF and (**b**) NaOH-CMDPF, adsorbent dosage level 30 ± 1 °C [C_0_ = 100 mg/L, sorbent dosage = 0.1–0.1 g, contact time = 4 h, pH = 6.0].

The effect of RDPF and NaOH–CMDPF dosage amounts for the removal of the phenol system was studied by varying the amounts in the range of 0.1–1.0 g in 100 mg/L phenol concentrations at the optimum pH of 6 at 30 ± 1 °C for 3 h. The RDPF and NaOH–CMDPF showed that by increasing the dosage amount, the removal percentage of phenol also increased (Figure 5a,b). As shown in Figure 5a,b, the phenol removal percentage was 81.1% at 1.0 g, which increased from 75.1% at 0.1 g. For NaOH-CMDPF, it was found to be 86.1% at 1.0 g, which increased from 80.2% at 0.1 g. To increase the dosage amounts of the RDPF and NaOH–CMDPF, more binding sites were made available surface for attachment, which in the end was responsible for the high removal percentage. On the other hand, with an increasing dosage amount of adsorbent, uptake capacity started to considerably decrease. A similar trend was observed for both the RDPF and the NaOH–CMDPF biomass because of the saturation and aggregation at binding sites. Moreover, above 0.6 g/L of RDPF and NaOH–CMDPF biomass, there was no enhancement in phenol pollutant removal efficacy.

### 3.4. Effect of Contact Time and Initial Concentration

**Figure 6:**
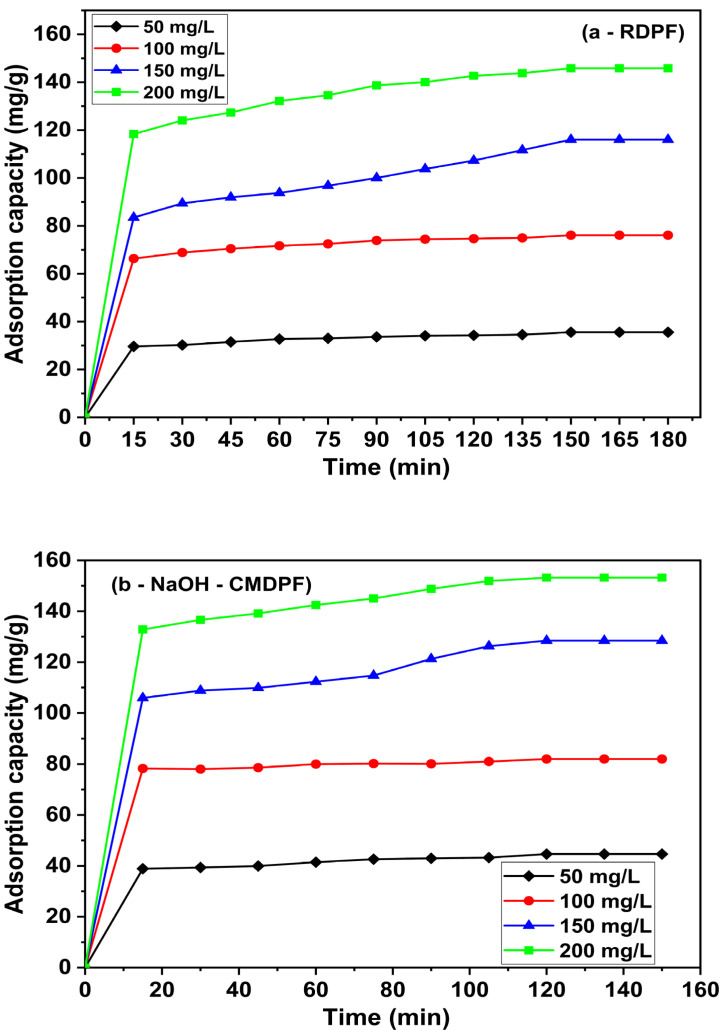
(**a**,**b**) Effect of phenol adsorption onto (**a**) RDPF and (**b**) NaOH-CMDPF, contact time 30 ± 1 °C [(◆) C_0_= 50 mg/L, (●) C_0_= 100 mg/L, (▲) C_0_= 150 mg/L, (■) C_0_= 200 mg/L; sorbent dosage = 0.1 g; contact time = 3 h, agitation rate = 175 rpm; pH = 6.0].

The effect of contact time and the influence of initial concentrations were significant parameters for phenol uptake removal efficiency by the adsorbents. The RDPF and NaOH-CMDPF agitation times were optimized from 15 to 180 min and 15 to 150 min at 30 °C, respectively. As represented in Figure 6a,b, adsorption capacity (*q_e_*) was enhanced rapidly with time and initial concentrations (50–200 mg/L) of both the RDPF and the NaOH-CMDPF biomass, respectively. Afterward, the removal efficiency of the RDPF and NaOH-CMDPF reached the equilibrium state at 150 and 120 min of contact time, respectively. The initial rapid uptake capacity at the beginning of adsorption was caused by the higher site availability and interacting groups of the external surface of the adsorbent. The RDPF and NaOH-CMDPF (Figure 6a,b) showed that adsorption capacity at equilibrium (*q_e_*) increased from 29.65 to 145.85 mg/g and from 38.87 to 153.19 mg/g, respectively, as the phenol initial concentrations were increased from 50 to 200 mg/L. Higher initial phenolic concentration inevitably led to a higher mass transfer driving force that was ultimately reflected in greater phenol uptake capacity. Thus, we kept the RDPF and NaOH-CMDPF agitation times at 150 and 120 min for our batch studies.

### 3.5. Adsorption Kinetics

The adsorption kinetic experiments were carried out to describe the phenol uptake rate and determine the residence time for the design of large-scale heterogeneous adsorption systems. The sorption of phenol was analyzed by different well-known kinetic models, e.g., pseudo-first-order (PFO; Equation (6)), pseudo-second-order (PSO; Equation (7)), Elovich kinetic model (EKM; Equation (8)), and intraparticle diffusion (IDM; Equation (9)).

The linearized PFO kinetic model can be mathematically expressed as follows [43]:

(6) $$log\left(q_{e}-q_{t}\right)=-\frac{k_{1}}{2.303}t+logq_{e}$$

The linearized PSO kinetic model can be mathematically expressed as follows [46]:

(7) $$\frac{t}{q_{t}}=\frac{1}{q_{e}}t+\frac{1}{k_{2}q_{e}^{2}}$$

Here, the rate constants of both PFO and PSO are presented by *k*_1_ (1/min) and *k*_2_ (g/mg/min), respectively. *q_e_* is the equilibrium uptake capacity (mg/g) and *q_t_* is the time-dependent sorption capacity (mg/g).

The adsorption rate constants of the PFO and PSO kinetic models along with correlation coefficients (R^2^) are shown in Table 2. Accordingly, the obtained Table 2 results show that the PFO model (R^2^) correlation coefficient values ranged from 0.757 to 0.992 and their experimental *q_e_*(exp) disagreed with the *q_e_*(calc) values. The PSO model (R^2^) correlation coefficient values ranged from 0.992 to 0.999 and their *q_e_*(calc) values were closer to *q_e_*(exp) values. Therefore, these results confirm the superior predictive capability of the PSO kinetic model compared to that of the PFO kinetic model in the present case of the biosorption of phenol using the RDPF and CMDPF.

The linearized Elovich equation can be expressed as follows [47]:

(8) $$q_{t}=\frac{1}{b}\ln t+\frac{\ln\left(ab\right)}{b}$$

Here, plotting $q_{t}$ against lnt yields a straight line with slope = $\left(1/b\right)$ and y-intercept = $\ln\left(ab\right)/b$. Parameter ‘a’ represents the initial sorption rate, while ‘b’ (g/mg) represents the surface coverage and activation energy during the chemisorption of the solute onto the adsorbent. It is evident from Table 3 that the predicted *q_e_* (calc) values did not show good agreement with their corresponding experimental *q_e_* (exp) values.

The intraparticle diffusion model (IDM) is mathematically described as follows [48]:

(9) $$q_{t}=k_{id}\sqrt{t}+C$$

where the slope $k_{id}$ (mg/g.min^1/2^) and y-intercept *C* (thickness of the boundary layer) can be evaluated from $\sqrt{t}$ versus $q_{t}$ plot. Both these values calculated from the IDM are shown in Table 3.

The PFO, PSO, IDM, and EKM model predictions and experimental data for the phenol concentrations varying from 50 to 200 mg/L (Supplementary Figures S7a–d and S8e–h) were compared. The outcome results show that kinetics data for the RDPF and CMDPF fitted well with pseudo-second-order reaction kinetics for the phenol system. Evidently, the PSO kinetic model best described the experimental data. The experimental results fitted with different kinetic models of phenol adsorption on the RDPF and CMDPF, and their Chi-square (χ^2^), regression coefficient (R^2^), and normalized standard deviation Δ*q_t_* (%) values are listed in Table 2 and Table 3. The RDPF and CMDPF of the PSO kinetic model regression coefficient (R^2^) values were greater than 0.992, which was greater than those of the EKM, IDM, and PFO kinetic models, and a similar trend was also found for the Chi-square (χ^2^) and Δ*q_t_* (%) data values. The experimental results clearly indicate that the PSO kinetic model provided a better fit for the biosorption of phenol on the RDPF and CMDPF.

### 3.6. Equilibrium Adsorption Isotherm Models

The equilibrium sorption isotherms played a significant role in the adsorption system. The adsorption isotherms provide critical information on the interactive process between the sorbate particles and the active surface sites on the adsorbent. In this study, three isotherm model parameters included the Langmuir, Freundlich, and Dubinin–Radushkevich isotherms.

The linearized Langmuir isotherm model can be expressed as follows [38,49]:

(10) $$\left(\frac{1}{q_{e}}\right)=\frac{1}{K_{L}q_{m}}\left(\frac{1}{C_{e}}\right)+\frac{1}{q_{m}}$$

where $q_{m}$ and $q_{e}$ are the monolayer biosorption capacity and phenol adsorbed per unit biosorbent mass (mg/g) at equilibrium, respectively. $K_{L}$ (mg/L) is the equilibrium constant, while the equilibrium concentration of the phenol in the solution is represented by $C_{e}$ (mg/L).

The adsorption on an energetically diverse adsorbent surface is described by the Freundlich isotherm model. Its generalized and linearized forms can be written as follows [50]:

(11) $$q_{e}=K_{F}C_{e}^{1/n}$$

(12) $$\ln q_{e}=\left(\frac{1}{n}\right)\ln C_{e}+\ln K_{F}$$

The Dubinin–Radushkevich isotherm model can be used to assess the energy of sorption and broadly classify whether the process of sorption is primarily chemical or physical in nature. The general linearized form of Equations (13) and (14) is represented as follows [51]:

(13) $$lnq_{e}=lnq_{m}-B\varepsilon^{2}$$

(14) $$\varepsilon =RT\;ln\left[1+\frac{1}{C_{e}}\right]$$

Here *ε*, *q_m_*, and *B* represent the sorption capacity (mg/g), Polanyi potential, and sorption-free energy per sorbate molecule constant (mol^2^/kJ^2^), respectively. The parameter *B* can be determined from the slope of the plot of $\varepsilon^{2}$ against $lnq_{e}$. The mean free sorption energy (E) per adsorbate molecule can be denoted as follows:

(15) $$E=\frac{1}{\sqrt{2B}}$$

A plot of $\varepsilon^{2}$ versus $lnq_{e}$. enabled the estimation of the isotherm parameters $q_{m}$ and E. This study assessed whether the sorption mechanism was physical adsorption (*E* < 8 KJ/mol), chemical adsorption (*E* greater than 16 KJ/mol), or ion exchange (8 < *E* < 16 KJ/mol) based on the correlation coefficients (R^2^), normalized standard deviations ($∆q\left(\%\right)$), and Chi-square values (χ^2^) for all three isotherms. Langmuir, Freundlich, and D-R parameter comparison values are represented in Table 4 and the predicted and experimental data are shown in Supplementary Figure S9a,b. Table 4 shows that, for both adsorbents (RDPF and NaOH-CMDPF), the calculated R^2^ values were highest for the D-R isotherm model (>0.996 and 0.995, respectively). This was followed by those of the Langmuir model, which were 0.998 and 0.998, respectively, and then those of the Freundlich model, which were 0.989 and 0.990, respectively. It was observed that the Langmuir and the D-R models best described the sorption of phenol onto the RDPF and NaOH-CMDPF adsorbent owing to the higher coefficient values of R^2^, lower Δ*q_e_* (1.21), and lower Chi-square (χ^2^) values (0.81) that were obtained from the determined parameters in the present study. The determined monolayer adsorption capacity values of different adsorbents employed for the removal of phenol as reported in different articles are matched with our present study results in Table 5.

## 4. Conclusions

The present study examined the capacity of the raw date palm fiber and NaOH chemically modified date palm fiber (RDPF and NaOH-CMDPF) agro-waste biomaterial for the eradication of phenol from aqueous wastewater. The biosorption process was affected by different factors such as the adsorbent dosage, pH, contact time, and initial concentration of the phenol. The adsorption process of phenol onto the RDPF and NaOH-CMDPF adsorbents was ideally and perfectly well-defined by using the Langmuir, D-R isotherm, Freundlich, and PSO models, with monolayer sorption capacities of 45.62 mg/g and 89.67 mg/g, respectively, at 30 ± 1 °C. The current study results confirmed that the RDPF and modified NaOH-CMDPF adsorbents can be employed as effective, inexpensive, and eco-friendly bio-adsorbents for the elimination of organic pollutants from industrial wastes as well as the purification of wastewater treatment plants. Clearly, the RDPF and NaOH-CMDPF can also be recommended for additional studies of the removal of high concentrations of phenol from aqueous contaminated wastewater.

## Figures and Tables

**Table 1:** Elemental data (C, H, N) analysis of RDPF and NaOH-CMDPF adsorbents.

No.	Sample Name	Carbon (%)	Hydrogen (%)	Nitrogen (%)
1	RDPF	46.13	5.98	0.05
2	NaOH–CMDPF	42.69	6.16	0.00

**Table 2:** PFO and PSO kinetic parameters of phenol on RDPF and NaOH-CMDPF.

**Phenol—Raw Date Palm Fiber (RDPF)**											
**PFO Kinetic Model**							**PSO Kinetic Model**				
**Conc (mg/L)**	***q*** **_e, exp_** **(mg/g)**	***q*** **_e, cal_** **(mg/g)**	***k*** **_1_** **(min^−1^)**	**R^2^**	**Δq_t_ (%)**	**χ^2^**	***q*** **_e, cal_** **(mg/g)**	***k*** **_2_** **(g/mg/min)**	**R^2^**	**Δq_t_ (%)**	**χ^2^**
50	35.56	7.78	0.015	0.992	85.49	1756.87	35.60	0.005	0.999	3.31	0.32
100	76.07	12.93	0.018	0.991	87.93	4816.63	76.67	0.003	0.999	2.11	0.28
150	116.03	47.32	0.014	0.889	71.72	1910.75	115.80	0.001	0.992	7.14	4.67
200	145.85	59.69	0.024	0.958	66.90	1835.26	149.57	0.001	0.998	4.27	2.21
**Phenol—NaOH Chemically Modified Date Palm Fiber (NaOH-CMDPF)**											
50	44.62	8.59	0.017	0.960	87.66	2114.67	44.55	0.006	0.999	3.30	0.32
100	82.01	5.47	0.014	0.888	96.33	17238.11	81.48	0.010	0.999	1.34	0.10
150	128.44	43.92	0.022	0.757	74.74	2052.09	129.34	0.001	0.992	6.04	3.09
200	153.19	41.67	0.027	0.858	78.94	3300.24	155.59	0.001	0.998	3.56	1.29

**Table 3:** IDM and EKM kinetic parameters of phenol on RDPF and NaOH-MDPF.

**Intraparticle Diffusion Model (IDM)**							**Elovich Kinetic Model (EKM)**						
**Phenol—Raw Date Palm Fiber (RDPF)**													
**Conc (mg/L)**	**q_e_, exp (mg/g)**	**q_e_, cal (mg/g)**	**k_id_**	**C**	**R^2^**	**Δq_t_ (%)**	**χ2**	**q(e, cal)** **(mg/g)**	**(1/b)ln(ab) (mg/g)**	**1/b (mg/g)**	**R^2^**	Δ **q_t_ (%)**	**χ2**
50	35.56	34.87	0.67	26.97	0.975	0.82	0.02	34.52	22.54	2.44	0.968	0.98	0.03
100	76.07	75.71	1.12	62.59	0.976	0.60	0.02	75.15	55.01	4.10	0.993	0.31	0.01
150	116.03	108.87	3.41	69.19	0.966	1.56	0.22	106.78	48.41	11.89	0.902	2.67	0.64
200	145.85	144.73	3.38	105.38	0.994	0.39	0.02	142.89	83.56	12.09	0.976	1.04	0.13
**Phenol—NaOH Chemically Modified Date Palm Fiber (NaOH-CMDPF)**													
50	44.62	43.37	0.77	35.38	0.946	0.96	0.03	43.03	31.36	2.50	0.893	1.37	0.05
100	82.01	80.77	0.46	75.96	0.868	0.48	0.01	80.55	73.61	1.49	0.800	0.59	0.02
150	128.44	122.48	2.98	91.93	0.865	2.12	0.37	120.93	77.66	9.29	0.770	2.76	0.63
200	153.19	150.64	2.95	120.38	0.980	0.60	0.04	149.31	105.13	9.49	0.926	1.18	0.14

**Table 4:** Isotherm parameters of phenol on RDPF and NaOH–CMDPF.

Phenol															
Adsorbent	Langmuir					Freundlich					Dubinin-Radushkevich				
	q_m_ (mg/g)	b (L/mg)	R^2^	Δ ***q*_*e*_ (%)**	χ^2^	K_F_ ((mg/g)(L/mg)^1/n^)	n	R^2^	Δ ***q*_*e*_ (%)**	χ^2^	q_s_ (mmol/g)	E (kJ/mol)	R^2^	Δ ***q*_*e*_ (%)**	χ^2^
**RDPF**	45.62	0.034	0.998	6.96	2.36	0.938	0.680	0.976	13.38	4.43	1.86	6.55	0.967	15.79	6.22
**NaOH-CMDPF**	89.67	0.033	0.999	7.87	2.28	2.465	0.779	0.990	8.09	3.54	2.35	7.08	0.985	10.01	3.26

**Table 5:** The maximum uptake capacities Q0 (mg/g) of different biomasses and the remediation process of phenol experimental conditions.

Adsorbent	Q^0^ (mg/g)	Experimental Conditions		References
		pH	Contact Time	
Macroalgae/alginate beads	9.5	6	120 min	[52]
Ziziphus leaves	15	6	300 min	[53]
Schizophyllum commune fungus	120	5	120 min	[54]
Spirulina and chitosan foam	447.6	6.5	120 min	[55]
Modified green macroalga	20	6	180 min	[56]
Pine cone powder	164.51	5	60 min	[7]
Trametes versicolor polyporus fungus	50	6	240 min	[57]
Sulphuric acid-treated pea shells, USAPS	125.77	7	180 min	[58]
Acid-treated pyrolytic tire char	51.92	6.6	60 min	[59]
Pine bark powder	142.85	6	120 min	[23]
Moroccan clay	15.11	4	180 min	[60]
Red mud	49.30	8	480 min	[61]
Guava tree bark	46.76	7	120 min	[62]
Neem leaves	74.90	3	240 min	[63]
Raw date palm fiber (RDPF)	45.62	6	150 min	Present Study
NaOH–CMDPF	89.67	6	120 min	

## Data Availability

Not applicable.

## Supplementary Materials

The following supporting information can be downloaded at https://www.mdpi.com/article/10.3390/ma16114057/s1.
