# Supplementary material for: Preparation, Characterization, and Chemically Modified Date Palm Fiber Waste Biomass for Enhanced Phenol Removal from an Aqueous Environment
Source: Materials (Basel). 2023 May 30;16(11):4057. doi: 10.3390/ma16114057 (PMC10254225; doi:10.3390/ma16114057)
Supplement: Supplementary file 1 [file materials-16-04057-s001.zip › materials-2368055-supplementary.pdf]

Supplementary Materials

Preparation, Characterization, and Chemically Modified Date Palm Fiber Waste Biomass for Enhanced Phenol Removal from an Aqueous Environment

Nadavala Siva Kumar [1,*], Mohammad Asif [1], Anesh Manjaly Poulose [2], Ebrahim H. Al-Ghurabi [1], Shaddad S. Alhamedi [1] and Janardhan Reddy Koduru [3]

[1] Department of Chemical Engineering, King Saud University, P.O. Box 800, Riyadh 11421, Saudi Arabia; masif@ksu.edu.sa (M.A.); ealghurabi@ksu.edu.sa (E.H.A.-G.); shaddadalhamedi@gmail.com (S.S.A)
[2] SABIC Polymer Research Centre, Department of Chemical Engineering, King Saud University, P.O. Box 800, Riyadh 11421, Saudi Arabia; apoulose@ksu.edu.sa (A.M.P)
[3] Department of Environmental Engineering, Kwangwoon University, Seoul 01897, Republic of Korea; redddyjchem@gmail.com (J.R.K)
* Correspondence: snadavala@ksu.edu.sa, (N.S.K); Tel.: (+966-537228108)

Legends to Supplementary Figures

1. **Figure S1.** Particle size distribution curve of 24 hrs ball-milled date-palm fiber (RDPF) adsorbent.
2. **Figure S2.** Particle size distribution curve of 30 min micro ball-milled date-palm fiber (RDPF) adsorbent.
3. **Figure S3.** N_2 adsorption–desorption isotherms for Raw date-palm fiber adsorbent.
4. **Figure S4.** N_2 adsorption–desorption isotherms for 24 hrs ball-milled date-palm fiber adsorbent.
5. **Figure S5.** N_2 adsorption-desorption isotherms for 30 min Micro ball-milled date-palm fiber adsorbent.
6. **Figure S6.** N_2 adsorption–desorption isotherms of NaOH-chemically modified date palm fiber adsorbent (NaOH–CMDPF).
7. **Figure S7(a–d).** Comparison of experimental and calculated values obtained from the PFO, PSO, intraparticle diffusion and Elovich kinetic models (RDPF).
8. **Figure S8(e-h).** Comparison of experimental and calculated values obtained from the PFO, PSO, intraparticle diffusion and Elovich kinetic models (NaOH–CMDPF).
9. **Figure S9.** Comparison of experimental and theoretical adsorption parameter values of (a) RDPF and (b) NaOH–CMDPF adsorbents from the Langmuir, Freundlich, D-R isotherm models.

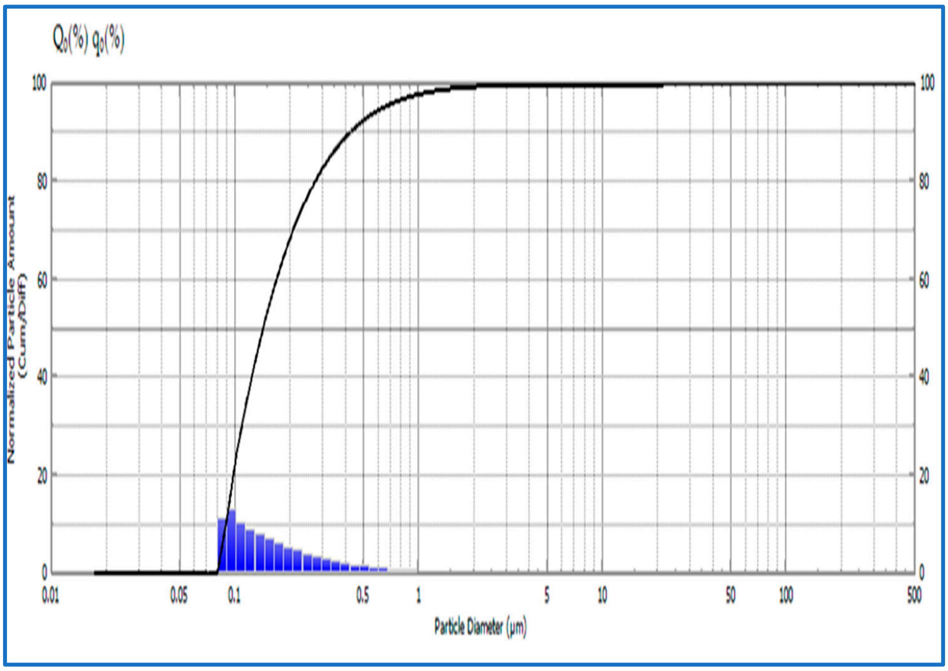

Figure S1.

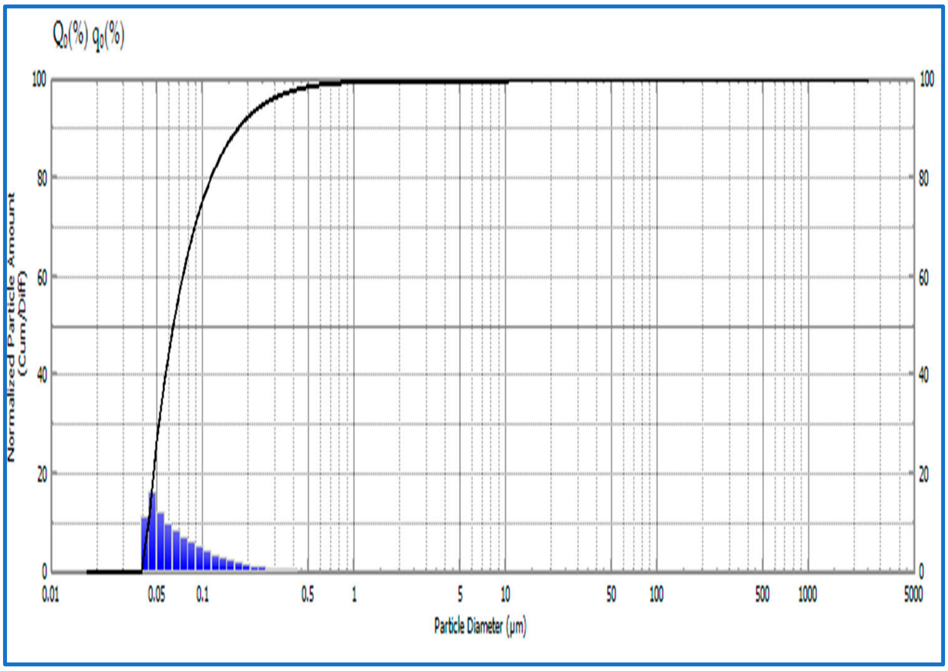

Figure S2.

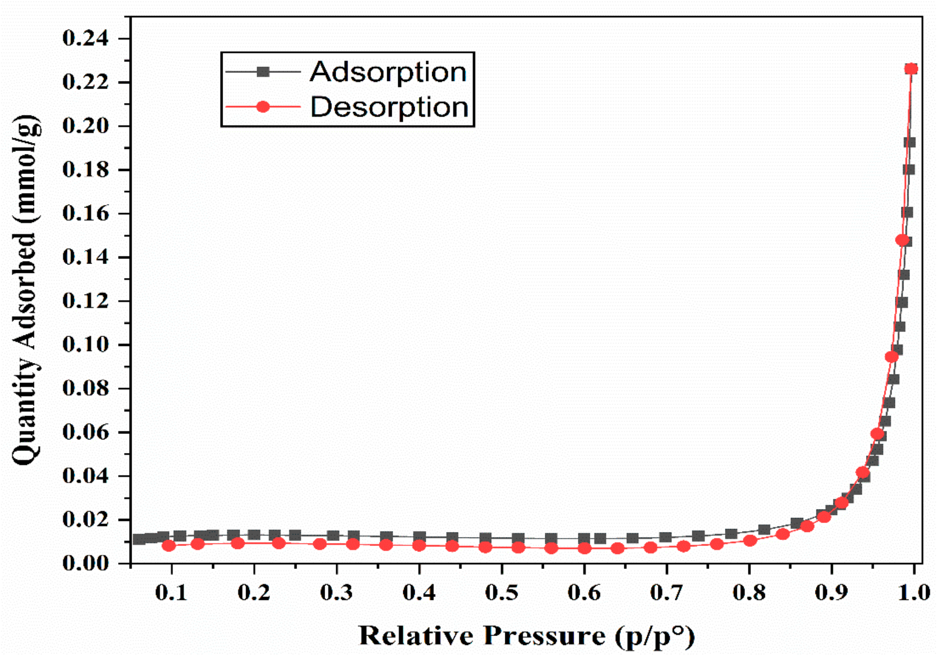

Figure S3.

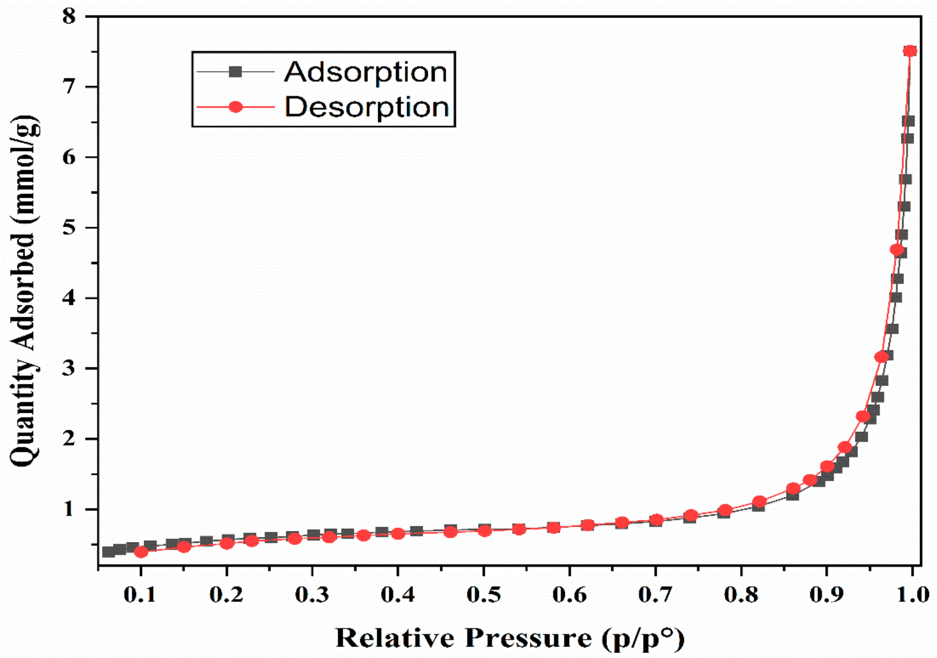

Figure S4.

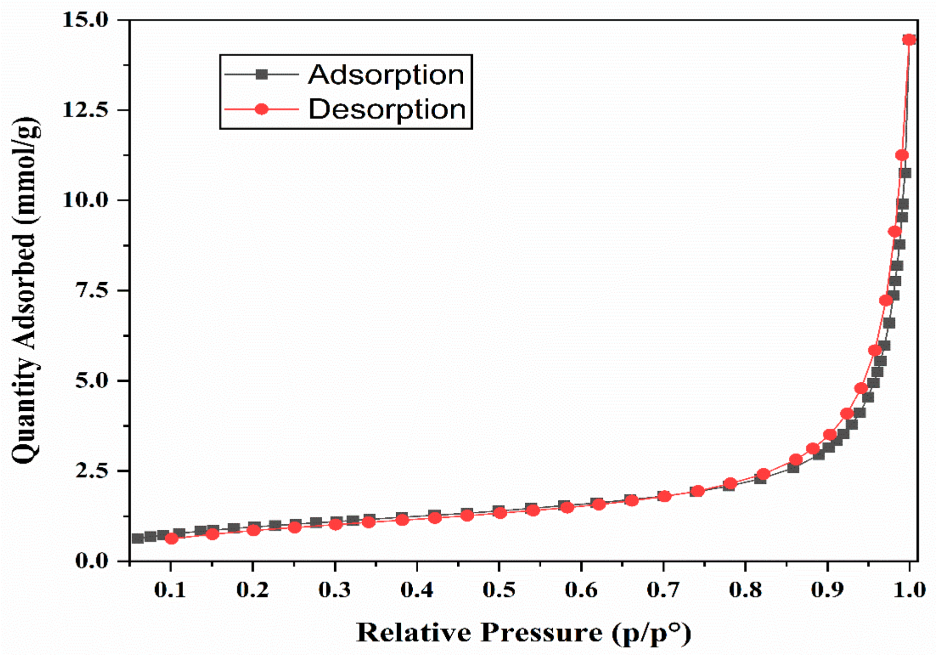

Figure S5.

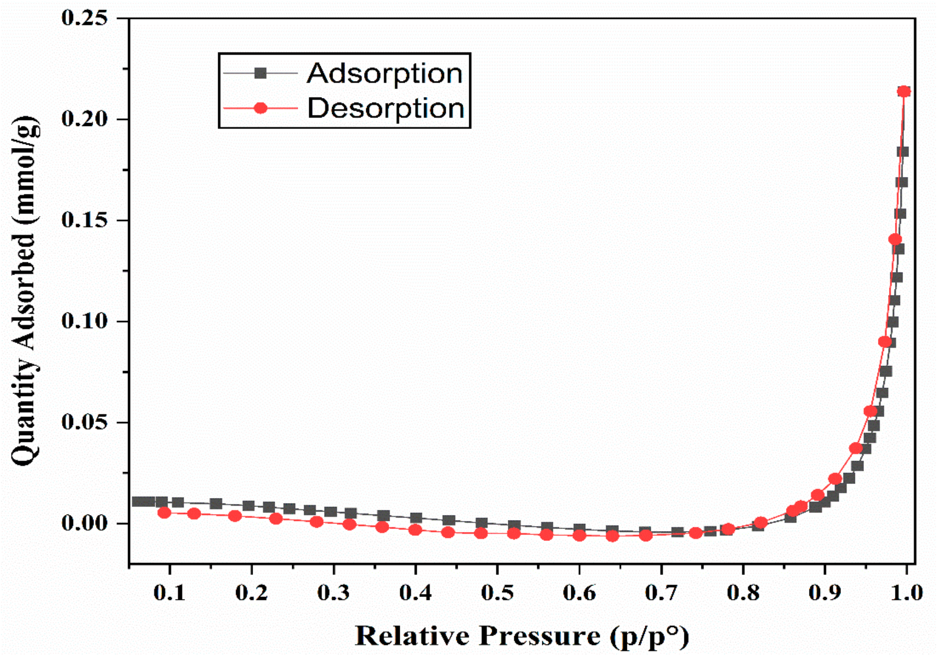

Figure S6.

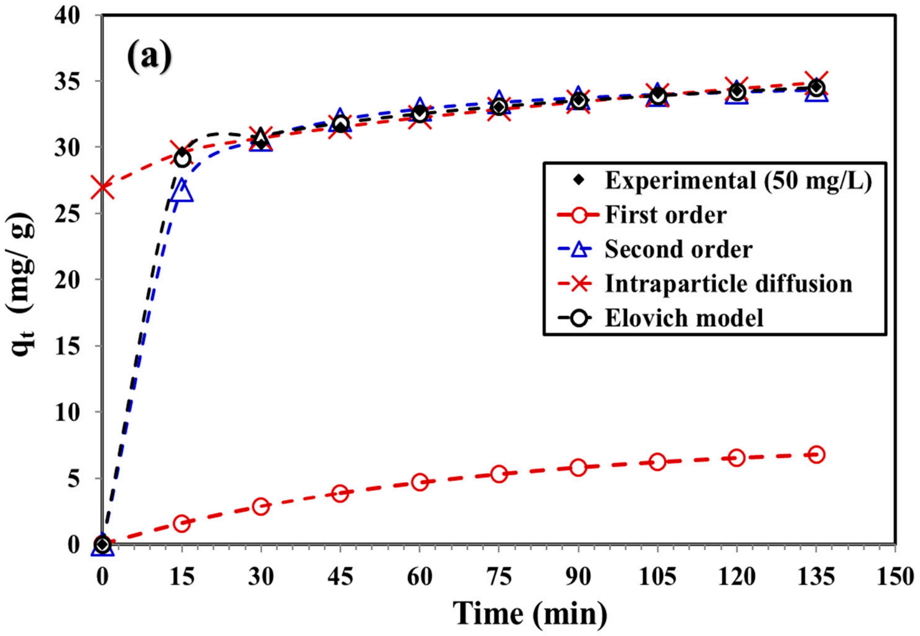
(a)
Experimental (50 mg/L)
First order
Second order
Intraparticle diffusion
Elovich model
q_t (mg/ g)
Time (min)

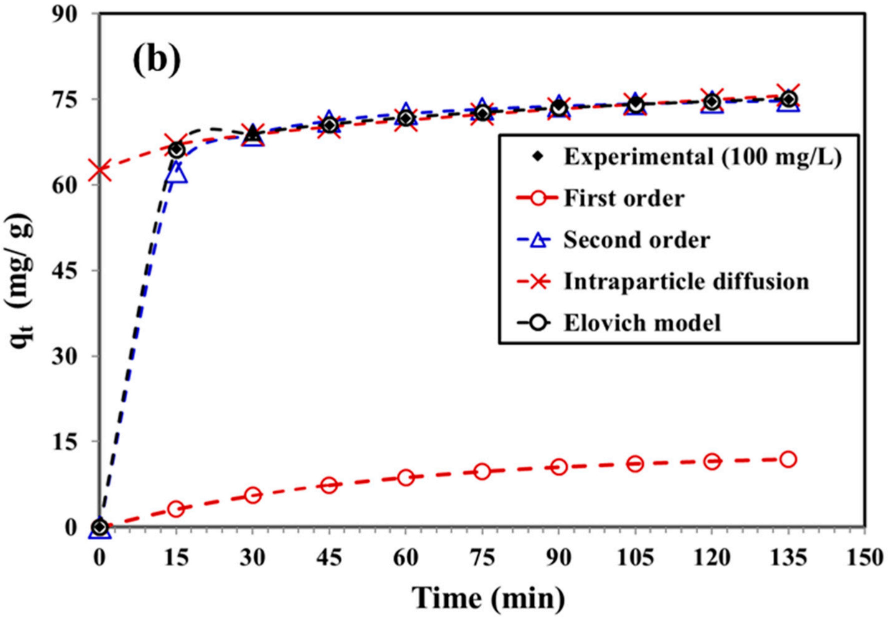
(b)
Experimental (100 mg/L)
First order
Second order
Intraparticle diffusion
Elovich model
q_t (mg/ g)
Time (min)

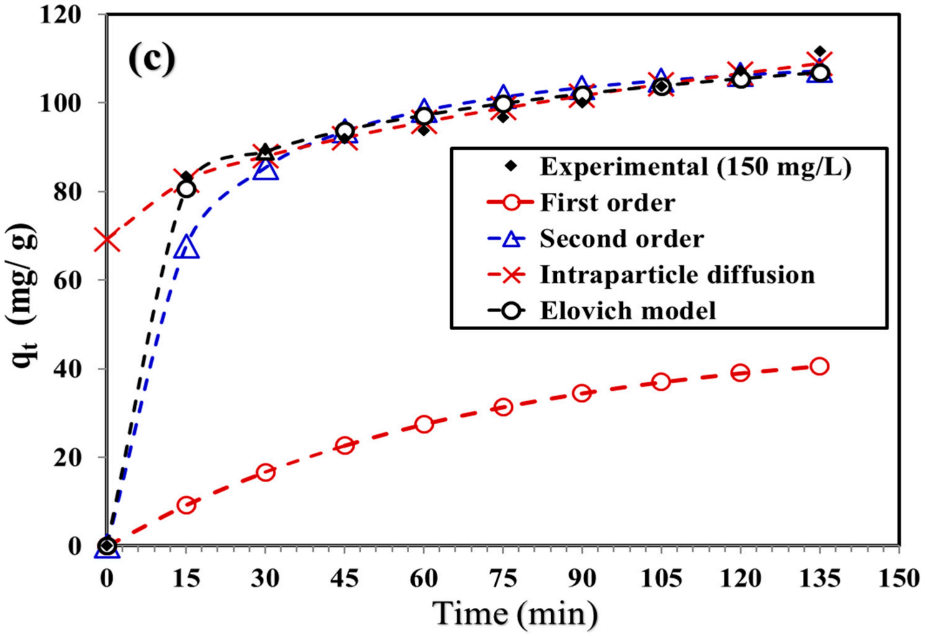
(c)
Experimental (150 mg/L)
First order
Second order
Intraparticle diffusion
Elovich model
q_t (mg/ g)
Time (min)
0
20
40
60
80
100
120
0
15
30
45
60
75
90
105
120
135
150

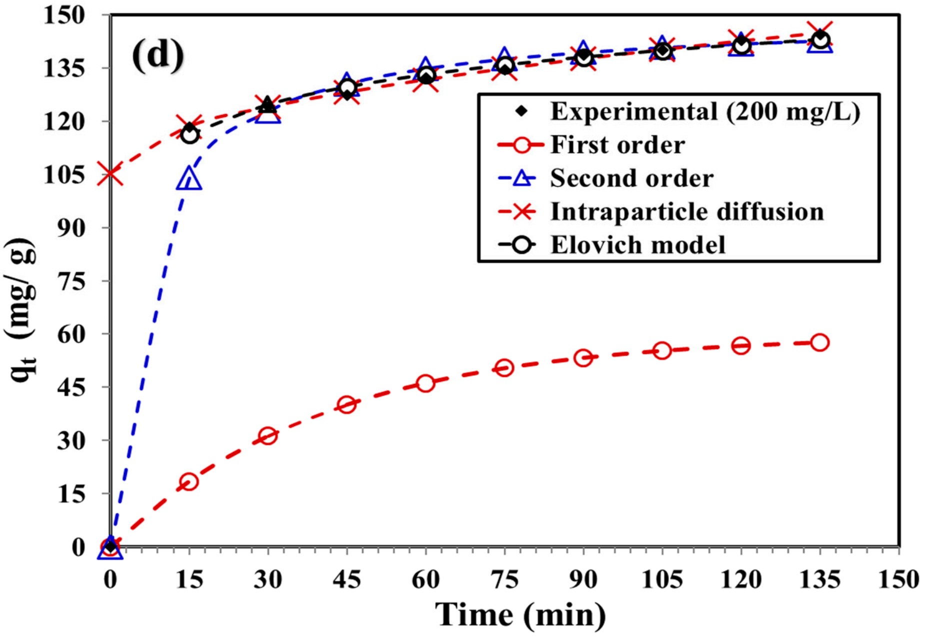
(d)
Experimental (200 mg/L)
First order
Second order
Intraparticle diffusion
Elovich model
q_t (mg/ g)
Time (min)
0
15
30
45
60
75
90
105
120
135
150
0
15
30
45
60
75
90
105
120
135
150

Figure S7 (a-d).

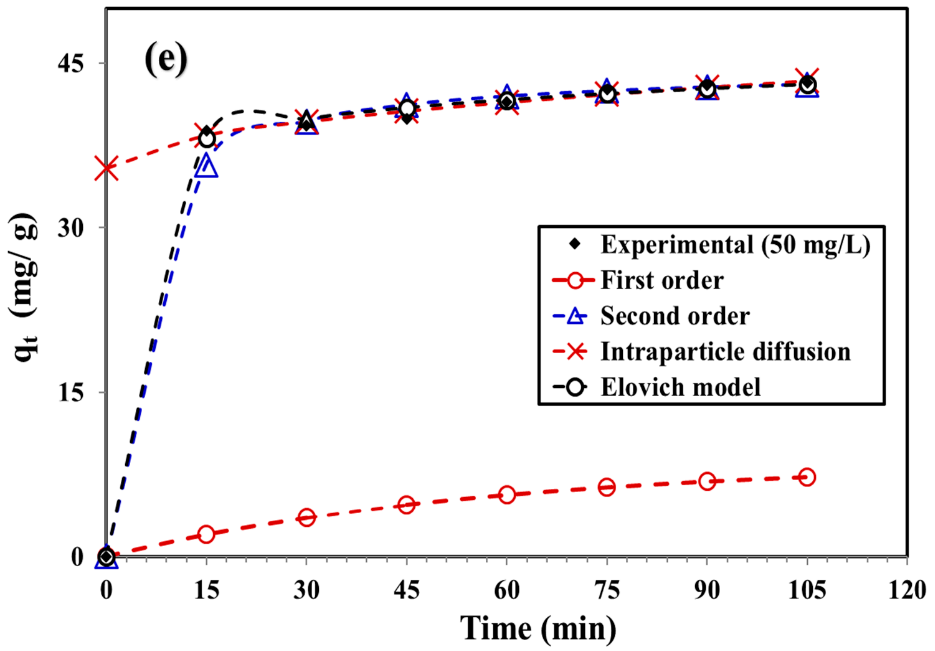

(e)
45
30
15
0
0
15
30
45
60
75
90
105
120
Time (min)
q_t (mg/ g)
Experimental (50 mg/L)
First order
Second order
Intraparticle diffusion
Elovich model

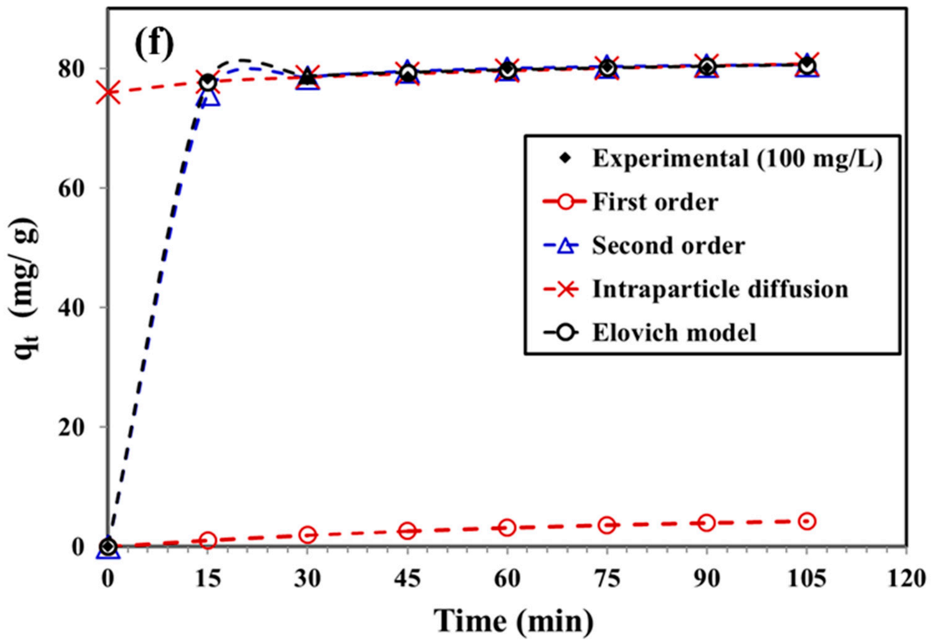

(f)
80
60
40
20
0
0
15
30
45
60
75
90
105
120
Time (min)
q_t (mg/ g)
Experimental (100 mg/L)
First order
Second order
Intraparticle diffusion
Elovich model

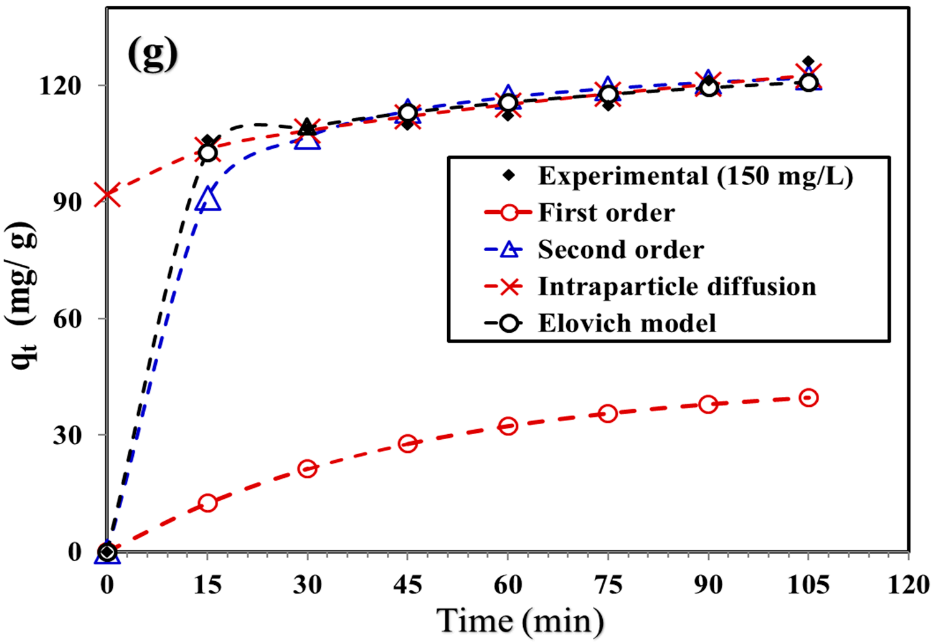
(g)
Experimental (150 mg/L)
First order
Second order
Intraparticle diffusion
Elovich model
q_t (mg/ g)
Time (min)
0
30
60
90
120
0
15
30
45
60
75
90
105
120

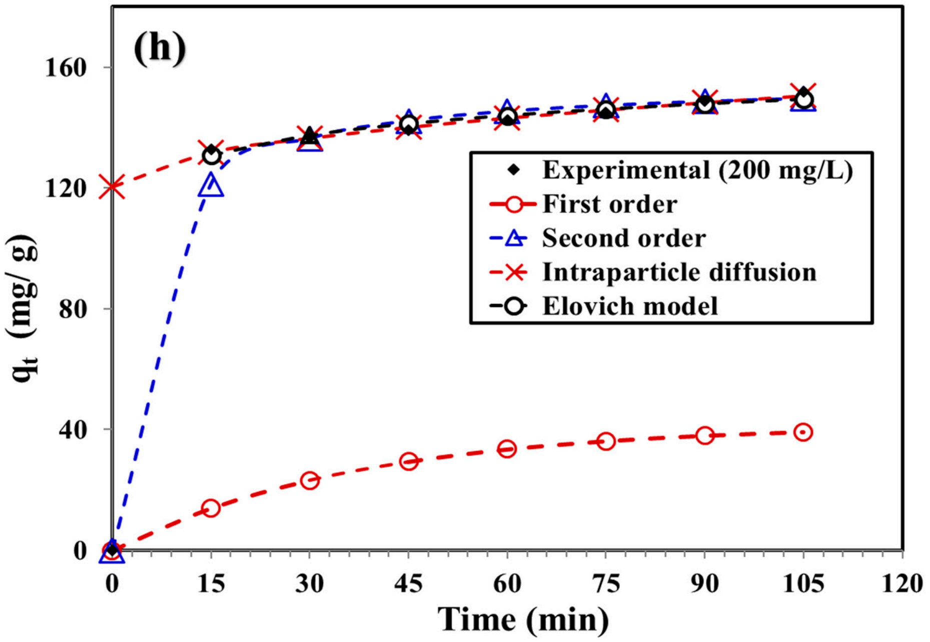
(h)
Experimental (200 mg/L)
First order
Second order
Intraparticle diffusion
Elovich model
q_t (mg/ g)
Time (min)
0
40
80
120
160
0
15
30
45
60
75
90
105
120

Figure S8 (e-h).

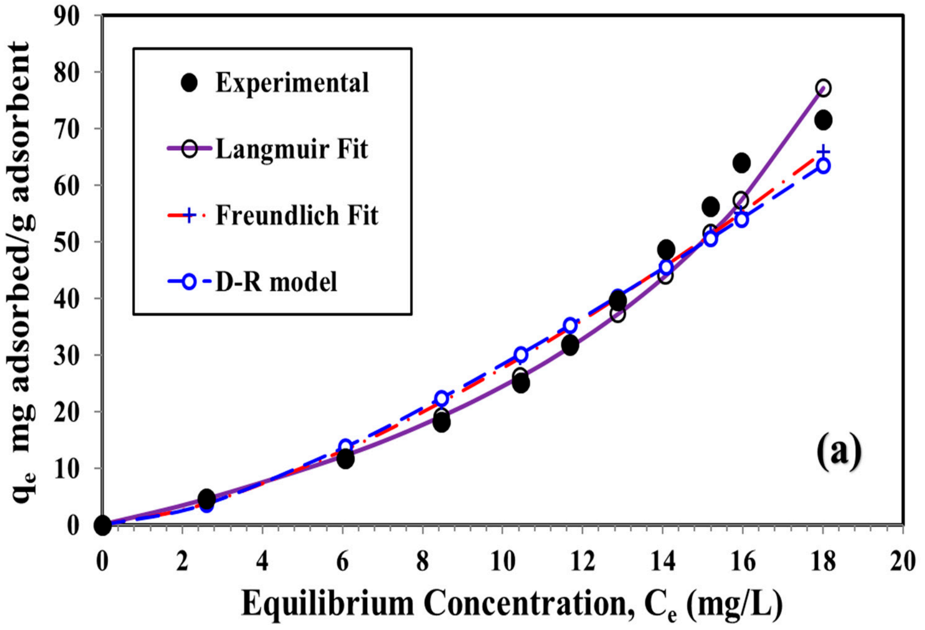

Experimental
Langmuir Fit
Freundlich Fit
D-R model
(a)
q_e mg adsorbed/g adsorbent
Equilibrium Concentration, C_e (mg/L)
0
10
20
30
40
50
60
70
80
90
0
2
4
6
8
10
12
14
16
18
20

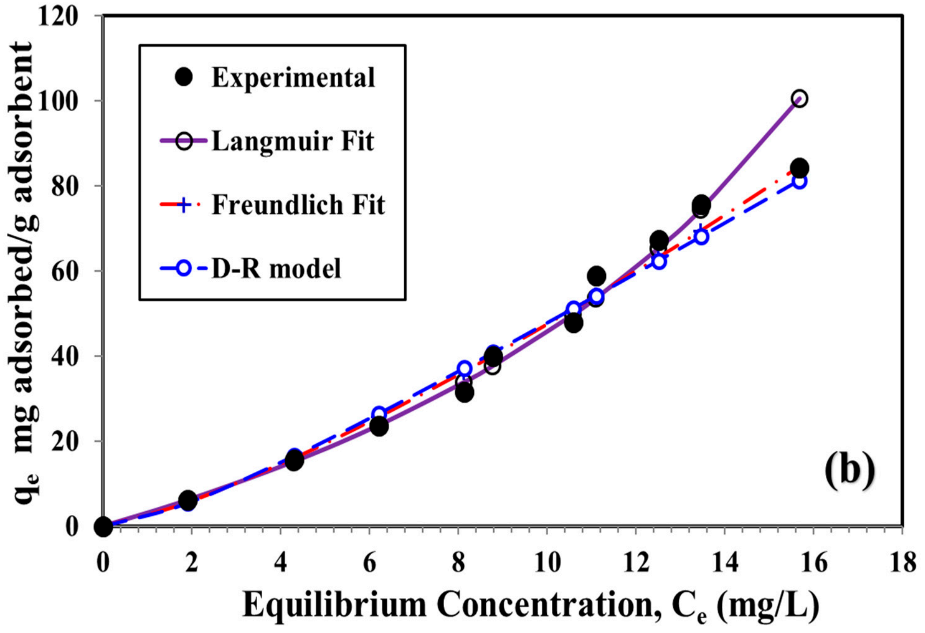

Experimental
Langmuir Fit
Freundlich Fit
D-R model
(b)
q_e mg adsorbed/g adsorbent
Equilibrium Concentration, C_e (mg/L)
0
20
40
60
80
100
120
0
2
4
6
8
10
12
14
16
18

Figure S9 (a-b).
